# Gathering Computational Genomics and Proteomics to Unravel Adaptive Evolution

**Published:** 2007-09-06

**Authors:** Agostinho Antunes, Maria João Ramos

**Affiliations:** REQUIMTE, Departamento de Química, Faculdade de Ciências, Universidade do Porto, Rua do Campo Alegre, 687; 4169-007 Porto, Portugal.

**Keywords:** bioinformatics, positive selection, molecular adaptation

## Abstract

A recent editorial in PLoS Biology by MacCallum and Hill (2006) pointed out the inappropriateness of studies evaluating signatures of positive selection based solely in single-site analyses. Therefore the rising number of articles claiming positive selection that have been recently published urges the question of how to improve the bioinformatics standards for reliably unravel positive selection? Deeper integrative efforts using state-of-the-art methodologies at the gene-level and protein-level are improving positive selection studies. Here we provide some computational guidelines to thoroughly document molecular adaptation.

The expression of the genetic information of living organisms depends largely on the functions of proteins. Important protein functionalities can be preserved by reducing genetic variability through purifying selection over long evolutionary time periods. In contrast, extensive genetic variation favoring amino-acid replacements in protein-coding genes through positive selection may originate novel functionalities. Understanding which gene is being influenced by natural selection can provide fundamental biological insight about species evolution and ecological fitness.

Selection can be inferred by comparing the rates of synonymous (silent; *d*_S_) and nonsynonymous (amino-acid replacement; *d*_N_) substitutions, where *d*_S_ < *d*_N_ is an indication of positive selection, and *d*_S_ > *d*_N_ suggests negative selection (Hughes and Nei, 1988). Powerful single-site analyses to detect selection have been developed (Yang and Bielawski, 2000) and have been implemented in relatively easy to use computer packages such as PAML (Yang, 1997). However, because these algorithms are so sensitive at detecting selection, many journals no longer publish papers that only use software such as PAML to identify adaptively evolving genes. Indeed, this issue was addressed by a recent editorial in PLoS Biology (MacCallum and Hill, 2006) where the editor points out the increasing number of articles claiming positive selection that have been recently published. To quote the above editorial “It is, therefore, no longer appropriate to sequence a gene in several species, stake a claim for positive selection, and expect the results to be published in a top-tier journal.” Such a policy is not limited to PLoS journals, but is also now being applied at more specialized journals such as Molecular Biology and Evolution, urging the need to improve evolutionary bioinformatics essays of molecular adaptation.

There are two main criticisms of single-site analyses. First, there is potentially a high probability of obtaining false-positives (Suzuki and Nei, 2002; Guindon et al. 2006). Second, a high *d*_N_/*d*_S_ ratio may not actually reflect a signature of selection, but result from demographic populations events (Kreitman, 2000) and non-neutral evolution at synonymous sites (Chamary et al. 2006). Regardless, the controversy around the topic of positive selection raises the question of how to improve the standards and statistics for reliable bioinformatics studies on positive selection?

Increasingly powerful computational genomics and proteomics tools may be the ultimate bridge between structural biology and molecular evolution. Many of the recent studies claiming positive selection have relied mostly on single-site analyses and the link with protein function, when addressed, relied mostly on the identification of potential selected sites in available crystal-structures, along with speculation about its functional importance. Clearly, complementary and deeper protein-level approaches, which have been mostly unexploited previously, are required. Indeed, recent studies have shown that protein evolutionary history can be largely retraced (Weinreich et al. 2006; Yoshikuni et al. 2006), suggesting that deeper integrative efforts using state-of-the-art methodologies at the gene-level and protein-level may significantly improve positive selection studies. Here we provide some computational guidelines to thoroughly document molecular adaptation.

First, single-site analyses (Yang and Bielawski, 2000) are useful for detecting selection at the gene level when it operates more or less constantly over evolutionary time, but are less useful when selection operates temporarily, as appears to occur for most biological innovations. Thus, second, recent methods, combining both gene and protein information, should be applied. The nature of the amino acid change (“conservative” or “radical” depending on the magnitude of the physicochemical difference between amino-acids; Smith, 2003; Woolley et al. 2003), and the physical location of aminoacid sites in the three-dimensional (3D) protein structure (Suzuki, 2004; Berglund et al. 2005) are important assets for deciphering and interpreting molecular adaptation. Moreover, rate-shift models are also useful for testing protein functional divergence (Knudsen and Miyamoto, 2001).

Third, molecular adaptations studies should apply protein-level analyses that can overcome some of the limitations of single-site methods (Suzuki and Nei, 2002; Suzuki, 2004). These include computational techniques such as molecular mechanics, quantum mechanics, and hybrid-methods that study biological systems in atomic detail, including enzyme mechanistic assessments and rational drug design (reviewed in Ramos and Fernandes, 2006). Homology-modeling is a reliable technique to computationally infer an unknown protein 3D-structure based on experimentally determined 3D-structure of a related protein (>50% amino-acid identity) (Martí-Renom et al. 2000). Even when tools such as *d*_N_/*d*_S_ fail to detect the selective history of a gene, a 3D-structural homology-model may detect non-negligible functional shifts (Andrés et al. 2004). Computational mutagenesis, molecular docking, and the calculation of electrostatics molecular potentials and free energies of association, reveal important functional interactions in enzymatic systems (complex ligand-receptor) and protein-protein interactions (Ramos and Fernandes, 2006). The implementation of such techniques using distributing computing and grid computing solutions may have great potential for future protein-level analyses at a genome-wide level.

Genomics and proteomics are rapidly-evolving research fields and their rational integration with other disciplines such as ecology and evolution has the potential to provide new perspectives on the process of adaptation relevancy and the neutral theory (da Fonseca et al. 2007; Marques et al. 2006). Rigorous interpretation and functional validation of targeted genes under adaptive evolution using integrated gene-level and protein-level information will improve the standards of reliable detection of positive selection and will be necessary to understand these fundamental evolutionary processes.
